# Leveraging community health workers as vaccinators: a case study exploring the role of Malawi’s Health Surveillance Assistants in delivering routine immunization services

**DOI:** 10.1186/s12960-023-00827-3

**Published:** 2023-05-31

**Authors:** Rebecca Alban, Emily Gibson, Jenny Payne, Tafwirapo Chihana

**Affiliations:** 1 VillageReach USA, 210 S Hudson St Suite 307, Seattle, WA 98134 USA; 2 VillageReach Malawi, PO Box 31348, Lilongwe 3, Malawi

**Keywords:** Community Health Workers, CHWs, Vaccination, Immunization, Community, Task-shifting, Malawi

## Abstract

**Background:**

Global chronic health worker shortages and stagnating routine immunization rates require new strategies to increase vaccination coverage and equity. As trained, trusted members of their local communities, community health workers (CHWs) are in a prime position to expand the immunization workforce and increase vaccination coverage in under-reached communities. Malawi is one of only a few countries that relies on CHWs—called Health Surveillance Assistants (HSAs) in Malawi—to administer routine immunizations, and as such offers a unique example of how this can be done.

**Case presentation:**

We sought to describe the operational and programmatic characteristics of a functional CHW-led routine immunization program by conducting interviews with HSAs, HSA supervisors, ministry of health officials, and community members in Malawi. This case study describes how and where HSAs provide vaccinations, their vaccination-related responsibilities, training and supervision processes, vaccine safety considerations, and the community-level vaccine supply chain. Interview participants consistently described HSAs as a high-functioning vaccination cadre, skilled and dedicated to increasing vaccine access for children. They also noted a need to strengthen some aspects of professional support for HSAs, particularly related to training, supervision, and supply chain processes. Interviewees agreed that other countries should consider following Malawi’s example and use CHWs to administer vaccines, provided they can be sufficiently trained and supported.

**Conclusions:**

This account from Malawi provides an example of how a CHW-led vaccination program operates. Leveraging CHWs as vaccinators is a promising yet under-explored task-shifting approach that shows potential to help countries maximize their health workforce, increase vaccination coverage and reach more zero-dose children. However, more research is needed to produce evidence on the impact of leveraging CHWs as vaccinators on patient safety, immunization coverage/vaccine equity, and cost-effectiveness as compared to use of other cadres for routine immunization.

## Background

Global routine immunization (RI) coverage stagnated beginning in 2010 [1] and then declined during the COVID-19 pandemic [2], with coverage dropping from 86% in 2019 to 81% in 2021 [3]. This leaves 25 million children with insufficient protection from routine immunization [3]. Providing routine immunization requires health workers who are available and adequately trained to administer vaccines. Most low- and middle-income countries (LMICs) face chronic health worker shortages that hamper service delivery for primary health care (PHC), including RI. The World Health Organization (WHO) estimates the world needs 10 million more health workers by 2030, primarily in LMICs [4]. Malawi faces significant health workforce shortages, with only 1.48 health workers per 1,000 population, their human resources for health are far below the recommended WHO targets. and Malawi faces health workforce shortages of 48% against its national targets [5].

In response to the health worker shortage, WHO views task shifting as “a viable solution for improving health care coverage by making more efficient use of the human resources already available” [6]. Research has demonstrated that task shifting, including the use of community health workers (CHWs) to deliver care, can improve population health [7].

As trained, trusted members of their local communities, CHWs are in a prime position to expand the vaccinating workforce and increase vaccination coverage in under-reached communities. This approach is in line with the latest Gavi Immunization Supply Chain (iSC) strategy, which aims to expand the reach of current vaccine supply chains to serve underserved and zero-does populations [8]. In many countries, CHWs currently play a key role in demand generation for primary health services, including RI [9], and could serve as a powerful bridge between remote or under-reached populations and the primary health care system. However, CHWs are rarely enlisted to administer vaccines. There is well-documented evidence that CHWs can successfully administer injectable medication [10] and already administer injectable contraception in many countries. In some countries, CHWs administer injectable contraceptives, often as part of a community-based family planning program in hard to reach areas either at health posts and through outreach services. Depending on country-specific policy, some CHWs can provide eligibility screening and initiate the first dose of injectable contraceptives along with subsequent doses, while others may provide the second and subsequent doses [11, 12]. In previous research, we identified that only 20 countries globally have leveraged CHWs to administer vaccines, but systematic documentation of where and how CHWs are administering vaccines is limited [13].

Leveraging CHWs as vaccinators for RI is an under-utilized task-shifting approach that could significantly expand the immunization capacity of the strained global health care workforce [13]. Further exploration of how to successfully leverage CHWs as vaccinators and the contextual factors that may facilitate or hinder success will help other countries consider if or how they might expand CHWs’ role in vaccination. Some of the key questions that potential adopters of this task-shifting may ask include: *What strategies and approaches work best to train, supervise, and equip CHWs with vaccine supplies? How and how is vaccine safety maintained? How do community members respond to receiving vaccines from CHWs?* As one of only a few countries with an established program of CHWs administering routine vaccines, Malawi presents a unique opportunity to describe the operational and programmatic characteristics of a functional CHW-led RI program.

The objective of this case study is to provide an in-depth description of a CHW-led routine immunization program. To our knowledge, no other peer reviewed articles have addressed a similar objective. This detailed account of how CHWs are used as routine vaccinators in Malawi provides insights on the successes and challenges of using CHWs as vaccinators, as described by key stakeholders working within the program and community members it serves.

## Case presentation

### Overview of research methods

From 8 to 27 March 2022, a Malawi-based research team conducted 36 semi-structured interviews in six districts, purposively selected to include a mix of rural and urban areas To ensure variety in the sample, we targeted one “high-performing” and one “low-performing” district in each of the country’s three regions based on routine immunization coverage. Rumphi in the north, Lilongwe in the centre and Chikwawa in the south emerged as districts with a higher immunization coverage rate, while Mzimba north, Dowa and Neno had lower immunization coverage rates [14]. Besides the immunization rates, rates of poverty also define the different regions, while Dowa (65.1%) and Chikwawa (61.2%) having significantly higher poverty rates compared to Rumphi (26.4%) and Mzimba (25.1%) [15]. Convenience sampling was used to select interview participations. Upon entering each district, the research team consulted with the corresponding Assistant Environmental Health Officer (AEHO) who referred us to local HSAs, HSA Supervisors, and caregivers of children under five from the community based on availability and communication skills.

Interview questionnaires were developed by a team with deep expertise in community health, supply chains, and qualitative research. The questionnaire development was guided by the desire to highlight CHW and immunization program elements, as well as the national policy and supply chain logistics context. Questionnaire content is closely aligned to the *Community Health Worker Performance Measurement Framework* [16]. Prior to data collection, the interview tools were piloted in Chitedze Health Center in Lilongwe with a caregiver of a child under five, an HSA, and an HSA supervisor. We conducted interviews with 15 HSAs, 6 HSA supervisors, 12 community members and 3 officials from the MOH or nationally represented partner organizations involved in immunization. Interviews were conducted by two experienced community health data collectors who had undergone a 1 day training on the study objectives and protocol. Interviews were conducted in either English or Chichewa depending on the preference of the interviewee. All interviewees were asked for informed consent prior to beginning the interview. By the time data collection had concluded, we noted consistency and repetition across respondents, indicating that saturation was achieved (Table 1).

**Table 1:** Interviewee summary

	# of interviews conducted
Ministry of health officials	2
Immunization Partners	1
HSAs	15
HSA supervisors	6
Community members (caregivers of children under 5)	12
Total Interviews conducted:	36

All interviews were audio recorded, transcribed and translated into English (when conducted in Chichewa) by professional translators. All identifying information was removed from transcripts. Translations were reviewed by TC, a bilingual Chichewa/English speaking researcher. We used a deductive/inductive coding process, first creating an initial codebook based on topic areas from the interview guide, then adding additional codes when gaps were identified during the coding process. A team of three coders (RA, JP, and EG) conducted primary and secondary coding of four initial transcripts using Atlas.ti 8 and reviewed these for intercoder agreement. Transcripts were then divided and assigned to coders to individually primary code, with a secondary coder reviewing and adding only when requested by the primary coder. Reports were then generated according to topic area and each coder was assigned 2–3 reports to review and summarize. All authors reviewed the summaries by topic area, and key takeaways were identified and agreed upon through discussion.

### Malawi immunization context

The primary government-employed CHWs in Malawi are known as health surveillance assistants (HSAs). The HSA program in Malawi began in the 1950s with HSAs serving primarily as vaccinators. Malawi has steadily increased HSA responsibilities over time; HSAs’ tasks now include administering routine immunizations, infant and child growth monitoring, providing health education, promoting sanitation, promoting Vitamin A supplementation, assessing and treating children with common illnesses, conducting home pregnancy and post-natal visits, and administering oral and injectable contraceptives, among other responsibilities [17]. HSAs are full-time CHWs and receive salaries on the government payroll. HSAs are unique amongst other CHWs around the globe, because they serve as the principal vaccinator cadre in the country and are responsible for administering the majority (an estimated 80%) of routine vaccinations in Malawi, along with COVID-19 vaccines [18]. Malawi’s immunization program is one of the most successful in the Africa region, consistently sustaining higher rates of DPT coverage than neighboring countries in central and southern Africa [19, 20]. Unlike many LMICs, Malawi’s vaccine coverage is higher in rural compared to urban communities (77% and 70%, respectively [19]), which may be linked to HSAs’ unique ability to reach hard-to-reach populations with immunization services. HSAs have been attributed with increasing vaccine acceptance and uptake of the human papillomavirus (HPV) vaccine introduced in Malawi in 2019 [21], and reaching Malawi’s prison populations with routine vaccines [22].

### Research findings

HSAs and supervisors described important advantages to having HSAs administer vaccines, including their relationships with and accessibility to community members. Most respondents viewed HSAs as trusted vaccinators and service providers. In reference to community trust in HSAs, one HSA shared:

*“[If] another person goes there with the vaccine, people will not receive it but if it is an HSA, they say our doctor has arrived.”*

Several respondents commented that use of HSAs as vaccinators was key to Malawi’s success in reaching relatively high levels of immunization coverage. When asked, government, partner, and HSA stakeholders interviewed all agreed that other countries should follow Malawi’s example and use CHWs to administer vaccines, provided they can be sufficiently trained and supported. Relieving nurses of vaccination duties helps maintain a more manageable workload for nurses and other health workforce cadres.

#### Vaccine administration

HSAs provide all standard EPI vaccines and were trained to administer COVID-19 vaccines. HSAs administer vaccines at both fixed sites such as hospitals or health centers and during outreach sessions in their communities. HSAs tend to conduct outreach sessions in teams of two to four, with responsibilities split up between team members. Outreach sessions occur at community locations including schools, churches, small shelters, and occasionally out in the open. HSAs described a clear understanding of their roles and responsibilities and reported vaccine administration as a core responsibility, though they also have many responsibilities outside of vaccination (Table 2).

**Table 2:** HSA responsibilities

Vaccination-related responsibilities	Responsibilities beyond vaccination
• Submitting requisitions for vaccines and supplies • Maintaining vaccine inventory and expiration records • Compiling and submitting vaccine reports • Planning and conducting vaccination sessions • Informing community members about vaccination • Reporting adverse events related to vaccination	Provision of routine preventive services • Administering and counseling for medication and supplements, such as ART, albendazole and vitamin A • Conducting under-five clinics • Participating in sexual and reproductive health programming, including educating pregnant women and administering family planning methods Health promotion • Conducting infectious disease prevention activities, such as for cholera and malaria • Coordinating youth-friendly programs • Conducting community education and social mobilization for various health initiatives • Supervising and participating in water and sanitation programs Other health systems roles • Conduct disease surveillance • Support disaster preparedness

#### Vaccine demand generation

HSAs are also tasked with RI demand generation activities, such as giving health talks to community members (some reported using tools, such as flip charts and posters), mobilizing community members for RI, and answering questions about vaccines. Most HSAs report providing counseling to caregivers related to their children’s immunizations, such as explaining the immunization schedule, how vaccines work, expected side effects, the benefits of vaccination, and dispelling myths about vaccination.

When asked about RI challenges, many HSAs cited difficulties related to demand generation activities, such as talking to caregivers who have doubts about routine immunization for their children. More recently HSAs have faced significant issues encouraging the uptake of COVID-19 vaccines. Most HSAs named demand generation and vaccination activities as their most time consuming activity.

#### Vaccine safety

To ensure potency of vaccines, HSAs are trained to routinely check vaccine vial monitors (VVM), monitor vaccine refrigerator temperatures, and use vaccine carriers or cold bags to maintain the cold chain when taking vaccines for outreach. HSAs are taught to always check the expiry dates and VVMs of their vaccines prior to administering them in facilities and prior to packing them up to take to the community. HSAs also play a central role in documenting and reporting adverse events following vaccination (AEFI). If an AEFI is identified, HSAs report them to the district using a standardized form and screen the patient for clinical follow-up care, if needed.

HSA supervisors enthusiastically agreed that maintaining vaccine safety was a significant part of their jobs, which they do through on-the-job coaching and direct observation, while HSAs vaccinate. Supervisors ensure that HSAs are following vaccine safety best practices, such as ensuring vaccine quality and potency, administering the vaccine correctly (correct syringe, correct amount, on the correct child), and discarding syringes and vaccine supplies safely.

#### HSA training

Before they are allowed to administer vaccines, all HSAs are required to attend a standardized HSA training administered by the Ministry of Health (MOH) and lasting between 8 and 12 weeks (the duration has evolved over the years). The training content is based on MOH guidelines that outline HSA roles and responsibilities, and thus includes other topics, such as family planning, TB and sanitation, but is heavily focused on immunization. HSAs estimated at least 50% of content was immunization-focused.

HSAs are assessed at baseline and endline, and given exams and practical exercises throughout the training. Whenever there is a new vaccine introduction or significant guideline change, HSAs said they participate in subsequent 1–2 day trainings or “briefings.” Table 3 lists training topics that were highlighted in key informant interviews.

**Table 3:** HSA training topics related to vaccination

• Pharmacology of how vaccines work
• How to conduct community vaccine education and outreach campaigns
• Vaccine storage best practices; concept of first expiry-first out (FEFO)
• Cold chain requirements by vaccine type
• Record keeping, including basic vaccine inventory management
• Vaccine administration methods (how and where on the body to inject)

Note this is not an exhaustive list of all training topics

Most HSAs agreed that their initial training was adequate to get them started on the job, but felt additional on-the-job learning to hone their skills was also critically important. Supervisors agreed that the initial training is comprehensive in terms of the theory provided, but emphasized that close supervision and support is important for new HSAs to develop practical skills. HSAs also overwhelmingly expressed a desire for more refresher trainings to keep their knowledge updated and adequately answer questions from their communities. One HSA described forgetting key vaccine information:

“*Sometimes we find ourselves forgetting, for example, how to vaccinate, how to store the vaccine, and how to know that this vaccine is damaged [or] expired. We need to always remember these things.”*

#### HSA supervision

Interviewees consistently described HSA supervisors as playing a crucial role in supporting HSAs to administer vaccines. HSA supervisors regularly observe HSAs vaccinating in health facilities and while doing outreach in communities, where they provide tips and feedback to HSAs, while they are on the job. Using a supervision checklist as a guide, they observe everything from how HSAs document their services provided and commodities used in reporting forms, to physical vaccine administration, to how HSAs interact with and answer questions from caregivers. Supervisors also take steps to provide quality control over the vaccination process by double checking vaccine expiry dates and VVMs before HSAs take vaccine supplies into the field, ensuring that the timing of the vaccine provision matches what is on the patient’s vaccination passport, and ensuring that the vaccine is injected in the right part of the patient’s body. Supervisors are also responsible for ensuring the availability of sufficient vaccine supplies for HSAs, collating HSA statistics on supplies and services provided, and facilitating relevant transportation for vaccine supplies and HSAs. Supervisors articulated that one of their primary challenges was lack of consistent transportation (Table 4):

“One of the challenges that I missed was review meetings, because we are supposed to be meeting these HSAs or these facilities at least once a quarter. …But then sometimes we do, sometimes we don't, based on the availability of resources…how should we address these challenges? I would propose that the DHO should be supplying us with enough fuel.” HSA Supervisor.

**Table 4:** HSA supervisor responsibilities related to vaccination

Supply chain:
• Ensure availability of vaccines for HSAs (place vaccine supply orders and manage inventory) • Organize transport for outreach visits • Provide oversight to ensure vaccine fridges are working, make alternative plans in event of a power outage, ensure cold chain is maintained from static to outreach clinics • Consolidate monthly reports from HSAs on services provided and commodities used Vaccine safety: • Observe HSAs vaccinating to make sure vaccines are administered correctly • Provide oversight on vaccine quality and potency checks, including checking VVMs and expiry dates • Ensure safety of HSAs while vaccinating (e.g., practicing injection safety, wearing masks, etc.) Admin/general management: • Provide performance feedback while observing HSAs • Hold monthly review meetings at the health facility with HSAs • Organize other supplies (e.g., uniforms, airtime, paper forms, boots, rain coats, backpacks, etc.) • Prepare work plans for HSAs • Maintain regular contact with HSAs and answer questions via phone/WhatsApp

#### Logistics and vaccine supply management

**Fig. 1:**
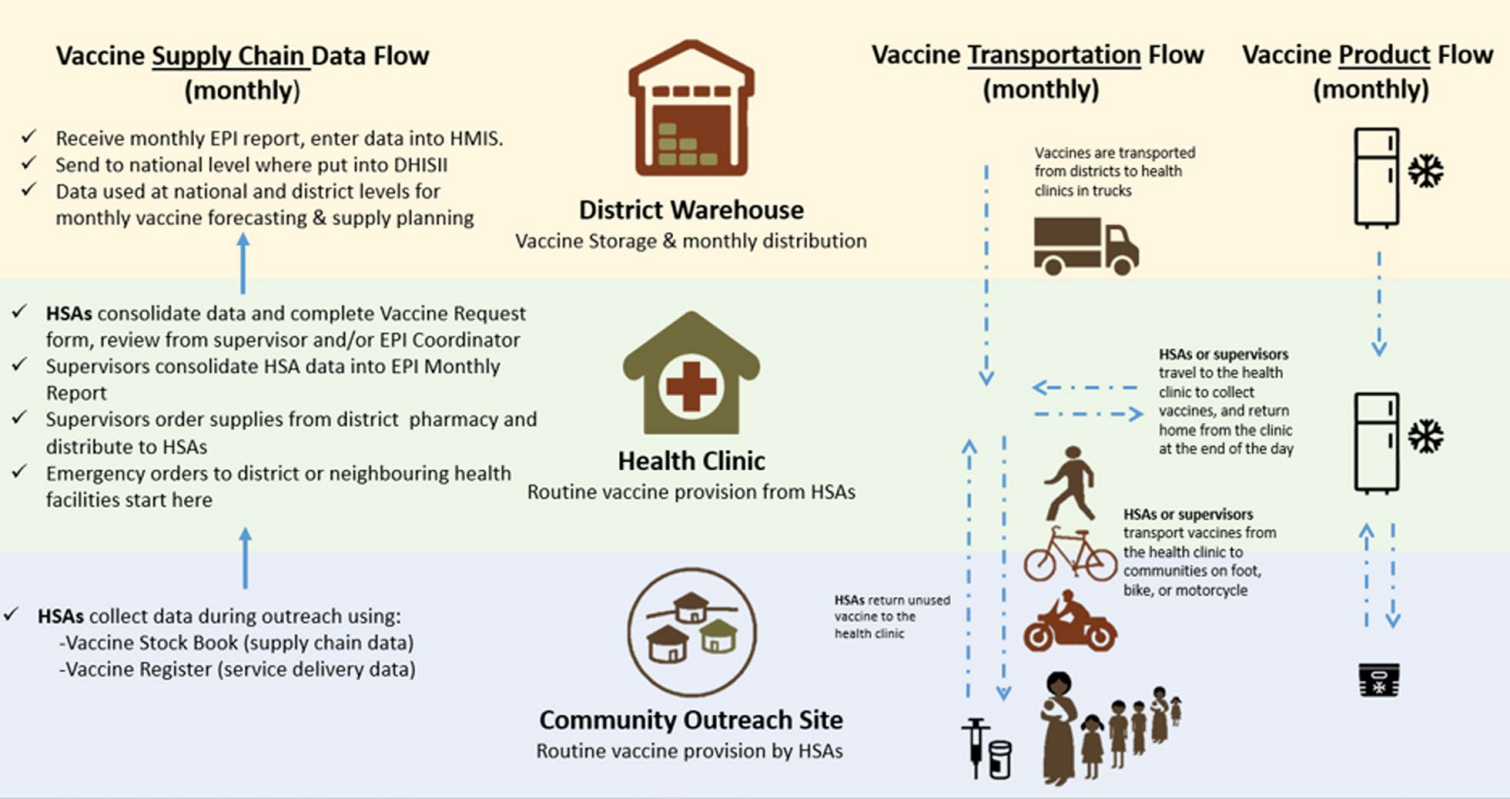
Diagram of the HSA vaccine supply chain

The vaccine supply chain in Malawi is parallel to that of other commodity groups, with separate transportation and reporting functions. Vaccine products are stored at the district warehouse, and distributed to health clinics on a monthly basis. The quantities distributed are based on a monthly report that consolidates inputs from HSAs on how many products were used and estimated needs for the following month based on each HSA’s target population. Figure 1 outlines the monthly downstream flow of vaccine products to HSAs, the transportation modes that are used, and the upstream flow of supply chain data from the community level to the district.

Although vaccine product stock outs were described as rare, there are some supply chain challenges, most commonly related to lack of reliable transportation. Vaccine supply orders can only be filled if there is fuel available at the district to deliver the supplies, otherwise HSAs must collect the order themselves or borrow from a neighboring health facility. HSAs frequently described placing emergency orders as a measure to avoid stock outs, indicating that the monthly ordering and reporting may not accurately predict true demand. Multiple HSAs commented that the unpredictable nature of how many people will show up to receive vaccinations at outreach sessions can also cause stock shortages.

HSAs and supervisors play an important role in maintaining the cold chain. HSAs regularly use cooler boxes and vaccine carrier bags to transport vaccines to and from community outreach sessions. Supervisors must ensure that the cold chain is maintained by monitoring fridge temperatures and electricity availability at the health facility, and verifying that HSAs transport vaccines appropriately. Approximately half of HSAs interviewed expressed a need for more vaccine carriers, especially to use during vaccine campaigns when HSAs are all deployed at once.

The MOH is responsible for providing transportation to deliver vaccines for HSAs to vaccinate in clinics and outreach sites, but HSAs and supervisors frequently mentioned major transportation challenges. Transportation issues and lack of fuel can delay vaccine distributions and limit the frequency of supportive supervision visits and review meetings. HSAs reported needing to travel long distances to pick up vaccine supplies and conduct outreach activities. HSAs might walk, use bicycles, motorcycles, government ambulances or other vehicles, or public transport when fuel is not available. One HSA said,

*“When we arrive, they will tell us they do not have fuel. But we have already told people that we will meet them at the clinic. We pay for the bicycle taxi, so we carry our vaccine, after arrival we administer the vaccine and then we carry back the remaining vaccine and return it. We use our money for expenses. We have used our money just because we want to save a Malawian child’s life.”*

#### HSA professional support

HSAs are salaried employees of the MOH, whose monthly payment is based on their professional grade and typically provided through banks in a reliable manner. Almost all interviewees felt the salary was too low, citing both the quantity of work HSAs do and inflation. Some reported receiving lunch allowances for community work, since it involves traveling to villages far from home, but immunization outreach was not included in this. Practices for reimbursement varied depending on the supporting partner, and HSAs described several unreliable methods and instances of never receiving promised reimbursement for participating in an activity. In addition, HSAs regularly have additional tasks added to their list of responsibilities without a corresponding increase in the workforce, consistent with what has been cited by several other studies on challenges faced by community health workforces [23]. COVID-19 provided an extreme example of this, as it added significantly to HSAs’ workload without any task shifting of other responsibilities. Supervisors lamented the lack of fuel and motorbikes to conduct supervision visits, and others noted the need for more bicycles, as well as gumboots, umbrellas and raincoats for the rainy season.

## Discussion

This research was successful in thoroughly documenting the operational and programmatic characteristics of Malawi’s CHW-led routine immunization program, as reflected in the *Community Health Worker Performance Measurement Framework* [16], as well as describe challenges and recommendations for other countries. Insights from HSAs, their supervisors, community members and MOH officials described a high-functioning vaccination cadre dedicated to reaching children with vaccines, though they face several challenges in day-to-day work requiring further attention.

Malawi’s HSA model provides an example of a system, where HSAs continue to participate in activities more routinely associated with CHWs (vaccine session planning, community education, etc.) and also administer vaccines, without needing to bring in additional cadres for support. In the Malawi context, HSAs provide the “full package,” enabling them to provide wraparound immunization services at fixed sites and outreach sessions with just members of their own health cadre, and not requiring accompaniment by nurses or other health workers (although occasionally they are present). Reducing the vaccination burden on nurses is a notable benefit of Malawi’s CHW-led model.

**Fig. 2:**
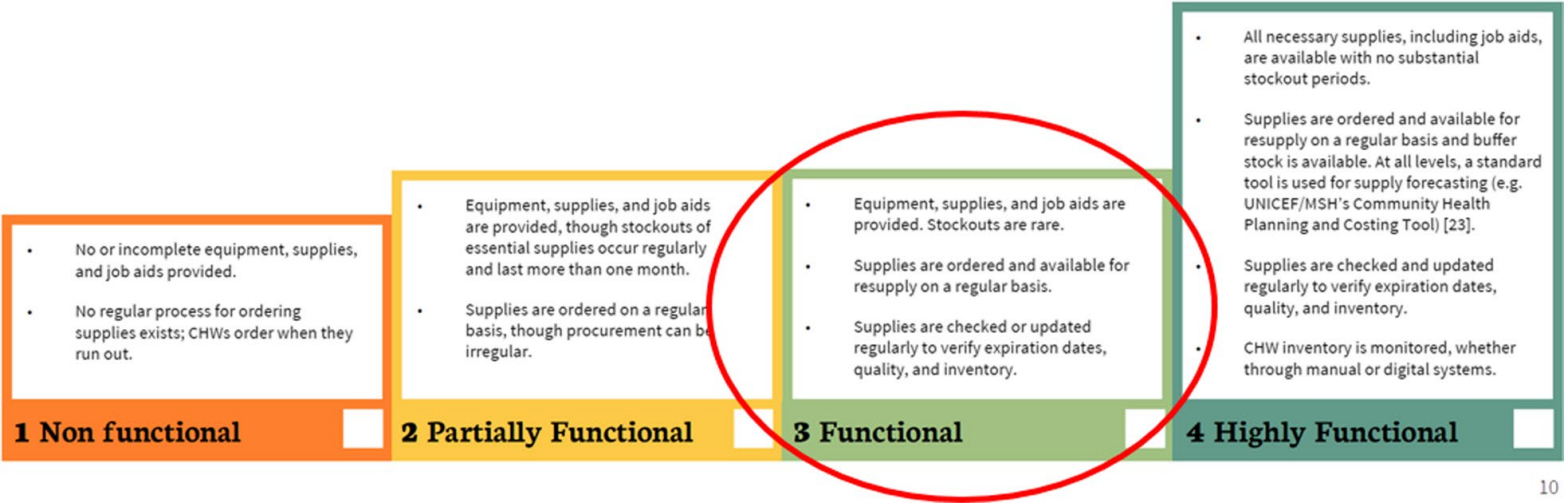
CHW AIM Tool: criteria for rating equipment and supplies availability for CHWs

In general, Malawi’s HSA program is consistent with the World Health Organization’s vision for a professionalized community health workforce [24], in that HSAs are paid salaries, supervised, and offered a standard competency-based training. HSAs described a strong understanding of best practices to ensure vaccine potency and quality, such as frequently checking expiry dates, checking VVMs, temperature monitoring, use of cooler boxes, and discarding open vials after appropriate number of days. Supervisors described that ensuring adherence to vaccine safety best practices was a key part of their role, and made an effort to directly supervise, while HSAs are vaccinating to review safety practices (Fig. 2).

Interviewees described the vaccine supply chain in Malawi as reliable, with infrequent vaccine stockouts at service delivery points, and extremely rare stockouts at district and national level. Using parameters set out in the Community Health Worker Assessment and Improvement Matrix (CHW AIM) tool [25], the vaccine supply chain can be rated as “functional”, because basic supplies are made available on a regular basis, but the system faces some challenges related to transportation and demand estimation that can cause vaccine shortages. Ensuring that the HSA vaccine supply chain continues to function at this level (or higher) is crucial to the success of Malawi’s CHW-led RI program.

Interviewees strongly emphasized that the primary advantages to having HSAs administer vaccines are their relationships with and accessibility to community members. Most respondents viewed HSAs as trusted vaccinators and service providers, and research indicates trust is a key factor in encouraging health behavior change, such as vaccine uptake [26]. When questioned about their perceptions of having HSAs administer vaccines, caregivers of children under five shared no hesitation or disagreement to receiving vaccination services from HSAs, indicating that this service delivery model is not negatively impacting demand or uptake of services.

Despite being an “institutionalized”, salaried CHW cadre, Malawi HSAs face similar challenges as other fledgling CHW cadres across Africa and Asia related to limited resources for training, supplies, and transportation [27]. The primary challenge of using HSAs as vaccinators described is the risk of inadequate training and the potential for poor quality work if not properly motivated and supported. Gaps in supportive infrastructure present challenges for HSAs and supervisors. Most HSAs and supervisors described their salary as insufficient given their growing workload and inflation, and modes of transportation have fallen into disrepair. HSAs frequently described using their personal resources for public transportation, and many described not having appropriate attire to conduct outreach sessions or travel to collect vaccines during the rainy season. Trainings, while thorough, are infrequent, leaving many HSAs with a desire for refresher sessions or reminders in between larger formal training events. Currently there are no routinely planned refresher trainings for HSAs, they are only conducted on an ad hoc basis when partners can provide funding, which results in many HSAs going for years with no training.

### Recommendations

Other countries considering involving community health workers in vaccination or transferring vaccination responsibilities entirely to CHWs can learn from Malawi’s experience. Of note, HSAs in Malawi report spending a significant amount of time administering vaccines and conducting vaccination-related activities. Other countries may not see similar results from involving CHWs as vaccinators if they are not equally dedicated to or focused on vaccination.

Other countries considering expanding the scope of CHWs in vaccine administration should pay close attention to the supportive infrastructure that enables HSAs to vaccinate effectively in Malawi. Specific recommendations include:

- Budget for transport and supplies: Plan to dedicate resources to ensure CHWs routinely have access to the infrastructure and supplies they require to administer vaccines outside of a hospital or clinic setting. This notably includes transportation to and from communities, fuel and maintenance for vehicles, and cooler boxes for last mile vaccine transport by CHWs. Likewise, supervisors require transportation to routinely conduct supervision visits. CHWs and supervisors may need additional equipment to travel for vaccination activities, such as raincoats.
- Conduct extensive foundational and routine training: In addition to ensuring a solid foundational training, countries should plan to provide CHWs with regular refresher trainings, conducted in-person or virtually, to help CHWs maintain their confidence and competence with vaccine administration information and skills.
- Coach and mentor CHWs using dedicated supervisors: Supervision is essential to ensure safe and successful vaccine administration, particularly when CHWs are new on the job. Supervisors should make time to directly observe CHWs administering vaccines, as well as CHW interactions with caregivers. As mentioned above, supervisors should be supported with adequate transportation resources to be able to conduct supervision visits.
- Provide CHWs with fair pay**:** Pay should be aligned to CHWs’ workloads (as recommended by the WHO [24]), in recognition of their valuable skills and contributions to national routine immunization coverage.
- Ensure a reasonable workload for CHWs: Routine vaccination is a core responsibility of HSAs in Malawi, which may be important to the program’s success. Other countries, whose CHW workforce currently do not vaccinate, will need to find strategies to incorporate this new responsibility into CHWs’ existing workplans and job descriptions. Possible strategies include: (1) CHW specialization (dedicating certain CHWs to focus on vaccination, while others maintain current roles), (2) further task shifting of current CHW responsibilities to other cadres, such as volunteers, and (3) expanding the current CHW cadre (retaining a higher number of CHWs). Consider these strategies to ensure CHWs can dedicate adequate time to conduct vaccination activities responsibly.
- Consider “task-shifting” and “task-sharing” approaches: *Task-shifting* and *task-sharing* are similar terms but refer to distinct approaches to leveraging CHWs for vaccine administration. Task-sharing would expand immunization service delivery to CHWs without removing it from other cadres [28], ultimately sharing the responsibility across multiple cadres. Task-shifting is the approach Malawi has taken, which concentrates service delivery duties among one cadre (HSAs) to alleviate the burden on nurses and expand health workforce capacity. Countries should consider the implications of both approaches before determining their own model for leveraging CHWs to administer vaccines.

This research had a number of limitations. First, HSAs have been the primary vaccinating cadre in Malawi for decades, which has given them many years to refine their approach, weave HSAs into the fabric of their community health and immunization processes, and become normalized in the minds of the general public. We are not able to speak to what the early years of the transition looked like or what it took to get to the point, where HSAs became trusted vaccinators. Second, data collected on safety and supply chain practices was entirely self-reported. We did not directly observe any HSAs administering vaccines or attempt to assess whether they follow procedures adequately. Likewise, we did not observe supervision, safety, or supply chain processes. Our use of convenience sampling to select interview participants could have introduced bias, because interviewees were referred by the District Associate Environmental Health Officer based on who were readily available and could communicate clearly, thus stakeholders who live in hard-to-reach areas or with poor communication skills may be under-represented.

## Conclusion

To our knowledge, this is the first in-depth documentation of how a national CHW cadre administers routine immunization. This account from Malawi showcases that national CHW cadres can successfully be trained, supported and consistently supplied with EPI products to provide routine immunization. This case study can inform other countries who may be interested in adopting this task-shifting approach. Leveraging CHWs as vaccinators is a promising yet under-explored task-shifting approach that shows potential to help countries maximize their health workforce, increase vaccination coverage and reach more zero-dose children, in line with the Gavi iSC strategy [8]. However, more research is needed to produce evidence on the impact of leveraging CHWs as vaccinators on patient safety, immunization coverage and equity, and cost-effectiveness as compared to use of other cadres for routine immunization.

## Data Availability

The data sets generated and/or analyzed during the current study are not publicly available to protect the identities of the research participants, but are available from the corresponding author on reasonable request.

## Abbreviations

AEFI: Adverse Event Following Immunization
CHW: Community Health Worker
COVID-19: Coronavirus disease
HAS: Health Surveillance Assistant
MOH: Ministry of Health
RI: Routine Immunization
VVM: Vaccine Vial Monitor
WHO: World Health Organization
